# Editorial: Application of network-theoretic approaches in biology

**DOI:** 10.3389/fgene.2023.1250548

**Published:** 2023-07-21

**Authors:** Mallana Gowdra Mallikarjuna, Manish Kumar Pandey, Rinku Sharma, Josh Clevenger, Sudeepto Bhattacharya

**Affiliations:** ^1^ Division of Genetics, ICAR-Indian Agricultural Research Institute, New Delhi, India; ^2^ The International Crops Research Institute for the Semi-Arid Tropics, Hyderabad, India; ^3^ Channing Division of Network Medicine, Brigham and Women’s Hospital and Harvard Medical School, Boston, MA, United States; ^4^ HudsonAlpha Institute for Biotechnology, Huntsville, AL, United States; ^5^ Department of Mathematics, Shiv Nadar University, Greater Noida, India

**Keywords:** gene regulatory network, co-expression analyses, network theoretic approaches, biology, protein–protein interaction, systems biology

## Introduction

Biological complexity explicitly occurs through non-linear interactions mostly entangled in nature. This complexity comprises many interactions among entities (*viz*., genes, proteins, metabolites, and species) at various spatial and temporal scales as complex adaptive systems showing characteristic features like self-organisation, modularity, emergence, non-linear interactions, collective response, and adaptation. The theory of complex networks provides an appropriate formal framework for modelling of such complex systems in order to obtain meaningful insights into biological complexity at the local or gene family level (Mallikarjuna et al., 2020; Mallikarjuna et al., 2022) and at the global scale (Sharma et al., 2021). The ocean of biological data generated by high-throughput technologies in the current genomics era have led to the application of various network-theoretic empirical investigations, in which the formal framework is used to obtain meaningful insights into system complexity. Our effort to pool studies on network-theoretic approaches in biology to the understanding of biological complexity has resulted in the compilation of ten research studies in the current Research Topic entitled *Application of network theoretic approaches in biology*, which are broadly categorised and highlighted under the following headings.

## Methods and tools

The availability of various user-friendly approaches and software applications has expanded the use of network-theoretic approaches to understand the complex biological process at individual and systems levels. Four articles in the current Research Topic deal with web tools and methods. A user-friendly web tool for the construction of gene regulatory networks, known as the “Consensus Approach for Gene Regulatory Network Construction” (CAGNC), was developed using the R programming language. CAGNC provides a network file with the edge scores representing significant interactions between each gene pair (Sarkar et al.). A new method, PolyReco, was developed to provide a reference model for processing, with automatic labelling of collinear regions and recognition of polyploidy events (Wang et al.). Lee et al. propose a hybrid intelligence platform, StarGazer (https://github.com/AstraZeneca/StarGazer), which provides an interactive dashboard that allows rapid searching for potential novel drug targets and the use of repositioning strategies via the Streamlit tool. Co-expression studies aid in the discovery of network patterns, functional module identification, and trait-linked marker mining at the system level. Finally, Xiao et al. present a knowledge-injected semi-supervised learning (KISL) method (https://github.com/Mowonhoo/KISL.git) for the identification of outstanding modules in a co-expression network. The KISL approach utilises *a priori* biological information and semi-supervised clustering to solve the issues present in contemporary clustering approaches, such as weighted gene co-expression network analysis (WGCNA).

### Plant and microbial systems

Two articles on plant systems are included in the current Research Topic, the first reporting on an investigation of chloride channels (CLCs) and the second presenting a study on the basic pentacysteine6 transcription factor. CLCs are known to regulate the pH of Golgi networks in plants. Here, an effort was made to identify the *CLC* gene family members in the recently sequenced wheat gnome (Fecht-Bartenbach et al., 2007). A total of 23 *CLCs* were identified in the wheat genome and exhibited a functional response to low-nitrogen and salt stresses (Mao et al.). Furthermore, genome-wide co-expression analysis in *Arabidopsis thaliana* indicated a key regulatory BPC6 regulating responses to various abiotic stresses (Zhang et al.).

In the domain of microbial systems, an agglomerative method consisting of complex network analysis and flux balance analyses (FBAs) was employed to examine the energy-intensive aromatic amino acid biosynthesis pathway (tryptophan, tyrosine, and phenylalanine) in 29 free-living bacteria and archaea species. The study identified several common hubs between the connected and the whole-genome networks, showing that the connected pathway network can act as a proxy for the whole-genome network in prokaryotes (Priya and Sinha).

### Human systems

At present, the utilization of network-theoretic approaches plays a significant role in unravelling intricate regulatory patterns and hubs within the fields of disease genomics and systems biology in humans (Barabási et al., 2011). In this Research Topic, one such study demonstrated the application of network-theoretic approaches to the identification of herbal medicines that act on immune cell infiltration and immune- and ferroptosis-associated gene expression levels to treat valvular atrial fibrillation. The study concluded that the herbs with rich curcumin content and resveratrol biochemical compounds (*viz*., Rhizoma Curcumae Longae and *Curcuma kwangsiensis*) mitigate myocardial fibrosis to improve valvular atrial fibrillation by modulating the TGFβ/Smad signalling pathway (Jiang et al.). Co-expression analysis is most widely employed to reveal highly synergistic sets of genes, functional modules, and hub genes at a systems level. Here, co-expression analysis of human *Candida* infection revealed the important modules and eight hub genes (*JUN*, *ATF3*, *VEGFA*, *SLC2A1*, *HK2*, *PTGS2*, *PFKFB3*, and *KLF6*) that were found to be enriched with hypoxia, angiogenesis, vasculogenesis, hypoxia-induced signalling, cancer, diabetes, and transplant-related disease pathways mediating host–pathogen interactions (Naik and Mohammed). Furthermore, co-expression studies with four molecular subtypes of breast cancer (*viz*., luminal A, luminal B, Her2, and basal) showed no correlations between copy number variations (CNVs) and the co-expression pattern of the genomic region 8q24.3 (Hernández-Gómez et al.).

## Conclusion and perspectives

In conclusion, our Research Topic presents various statistical methods and tools expanding the utility of network-theoretic approaches. Other articles demonstrate the application of various network approaches in developing our understanding of biological phenomena in plant, microbial, and human systems. Nevertheless, for large-scale applications and utilisation of network approaches in biology, there is a further need to undertake some of the following measures, although this is not an exhaustive list. First, more efforts should be made to develop biologist-friendly servers and tools for various types of network analysis, which can allow us to derive meaningful information from the ocean of *omics* data. Second, there is a need for the development of system-specific network approaches in order to understand species interactions from complex ecological and evolutionary perspectives. Finally, validation of major hubs through genetic and in-depth network-theoretic approaches could demonstrate the biological significance of network-theoretic studies in biology.
